# Bioelectrical Impedance Analysis to Increase the Sensitivity of Screening Methods for Diagnosing Cancer Cachexia in Patients with Colorectal Cancer

**DOI:** 10.1155/2020/3874956

**Published:** 2020-08-20

**Authors:** J. Szefel, W. J. Kruszewski, M. Szajewski, M. Ciesielski, A. Danielak

**Affiliations:** ^1^Division of Propaedeutics of Oncology, Medical University of Gdansk, Gdansk, Poland; ^2^Department of Oncological Surgery, Gdynia Oncology Centre, Gdynia, Poland

## Abstract

**Background:**

Currently used methods for detecting and monitoring cancer cachexia (CC) are not sensitive enough. In this field, there is a need to implement new instruments into clinical practice.

**Objective:**

Determining the usefulness of bioelectrical impedance analysis (BIA) for detecting and monitoring CC in patients with colorectal cancer (CRC).

**Methods:**

158 people were invited to the study (70 from CRC and 88 controls). Their body composition was determined using BIA, and their nutritional status was determined according to NRS 2002, SGA, and BMI criteria. For statistical data analysis, Student's *t*-test, Mann–Whitney *U* test, and AUC ROC were used.

**Results:**

Men with CRC stage I had higher values of FMI, SMMI, and ECW/TBW (*p* < 0.05) than in stages II–IV, and women with CRC stage I had higher values of FMI, FFMI, and FM/FFM than in the group of stages II–IV (*p* < 0.05). The ability of FFMI to detect malnutrition relative to SGA was low (sensitivity: women 40%, men 40% and specificity: women 74%, men 70%).

**Conclusions:**

SGA and NRS 2002 scales are dynamic and consider changes in nutritional status over time, while BIA is static and does not consider these changes. Therefore, BIA is not a good tool for screening nutritional status. BIA successfully identifies differences in body composition depending on cancer stage and advancement of CC. Therefore, after the diagnosis CRC, just to monitor the disease advancement and state of CC, it is worth comparing the results of periodically repeated BIA.

## 1. Introduction

The main cause of malnutrition is starvation, and cancer cachexia (CC) is caused by neoplastic factors and systemic inflammatory reaction [1, 2]. Despite many differences, the consensus of the Global Leadership Initiative on Malnutrition (GLIM) recommends the use of “cancer cachexia” to determine malnutrition in oncological patients [3]. GLIM has established five malnutrition criteria, three phenotypic and two etiological. The diagnosis of cancer meets the etiological criterion, but at least one of the three phenotypic criteria (nonvolitional weight loss—WL, low BMI, and reduced skeletal muscle mass—SMM) still needs to be met to diagnose CC.

Low BMI is effective for the recognition of refractory cachexia, but unreliable in earlier stages due to the prevalence of overweight (BMI > 25 kg/m^2^) and obesity (BMI > 30 kg/m^2^) [4, 5]. Thus, despite WL, many patients with CC will still be overweight [6]. The frequency of SMM loss in CC is variable. It has been established that the reduction of SMM covers from 5% to 89% of patients [7]. Due to the effects caused by the factors produced by cancer and the microenvironment of cancer, Fearon et al. described three stages of clinical relevance: precachexia, cachexia, and refractory cachexia [8]. The recognition of precachexia is difficult because the symptoms such as WL (less than 5%), anorexia, and impaired glucose tolerance are poorly expressed and proceed the loss of SMM. Finally, in refractory cachexia, management of WL is no longer possible due to active catabolism or presence of cachectic factors. At this stage, life expectancy is less than 3 months, and the goal of therapy is palliation of symptoms and reduction of stress.

Nutritional Risk Screening 2002 (NRS 2002) and Subjective Global Assessment (SGA) are easy to perform, but the results are often not reliable [9]. Arends et al. and Attar et al. demonstrated that the assessment of nutritional status by physicians in about 43% of patients was incorrect [10, 11]. Therefore, new, easily accessible instruments to diagnose and establish the stage of CC are being sought. They are expected to determine the body composition, severity of anorexia, physical fitness, and other features that justify the implementation of nutritional intervention. DXA is a reference method for body composition measurements, but BIA is cheaper and more easily available; moreover, it provides fast results and requires minimal operator training. Ræder et al. have shown that BIA measurement in most colorectal cancer (CRC) patients provides similar accuracy to DXA [12].

The indication for nutritional treatment is the diagnosis of CC and the determination of its stage, which is related to the loss of SMM and the change of proportions of other body compartments. Therefore, the aim of our study was to determine the effectiveness of BIA in relation to other screening tests. We chose patients with CRC because it is often accompanied by overweight or obesity, which effectively masks many symptoms of malnutrition.

## 2. Subjects and Methods

It was a prospective observational study carried out in the Department of Surgical Oncology, Polish Red Cross Maritime Hospital Gdynia Oncology Center according to the Declaration of Helsinki and its subsequent amendments. The study was approved by the Independent Bioethics Committee for Scientific Research at the Medical University of Gdańsk (Protocol Approval NKBBN/9/2015) and by the data protection officials at Polish Red Cross Maritime Hospital Gdynia Oncology Center.

### 2.1. Patients

158 people were included in the study (86 women, 54% and 72 men, 46%). They were informed about the methodology and purpose of the study and signed a consent form. The diagnosis of CRC (ICD-10 18–20) had to be proved by histopathological examination.

The group of patients with CRC, in stages I–IV according to TNM system, consisted of 70 people (36 women and 34 men). None of them had been previously treated oncologically. The control group consisted of 88 patients (50 women and 38 men), with no history of cancer and inflammatory or metabolic diseases. To balance the age-related effects of age-related sarcopenia on the BIA results, all study participants were divided into 2 groups: below and above 65 years of age [13]. The number of CRC cases increases rapidly after 50 years of age; therefore, to increase the reliability of statistical analysis results, people under 50 years of age were excluded from the study.

### 2.2. Measurements BIA

CT imaging, MRI, and DXA are accurate and preferred in scientific research. However, we have decided that BIA will be increasingly available for body composition screening.

The BIA seca mBCA525 device (seca GmbH and Co. KG) was used in the study. The measurements were carried out under standardized conditions according to the manufacturer's protocol, at 50 kHz. Patients were weighed in underwear and without shoes, and the height was measured in a standing position. BIA is a method for determining important body components (e.g., skeletal muscle mass index—SMMI, fat free mass index—FFMI, and fat mass index—FMI) for CC recognition. We also determined their proportions as the weight changes of these compartments are not synchronous [7, 14].

Most research on CC involves patients with tumors of different organs, although the cancer biology is different and depends on the location of the cancer type. Therefore, patients with one type of cancer were included in the study. We chose patients with CRC because of the often-accompanying obesity that makes it difficult to recognize and determine the stage of CC.

Patients excluded from the study were with

- Pacemakers (the current from the BIA device may disrupt their function)
- Abnormal body shapes (e.g., denture/implant orthopedic)
- Diseases that cause fluid retention (e.g., kidney failure, cirrhosis)
- BMI <16 and >35 kg/m^2^ (BIA results outside this range are incorrect) [15]

The height (meters) and weight (kilograms) of patients were obtained using standard procedures and indices (FMI, FFMI, and SMMI) were calculated by dividing the weight of a given body compartment by the square of height (meters^2^).

### 2.3. Nutritional Status

The nutritional status in NRS 2002 was evaluated by doctors admitting patients to the hospital, and the members of the research team assessed according to SGA. The results of the NRS 2002, SGA, and BMI assessments were simplified for statistical analysis. Patients were classified into one of two nutritional categories, respectively: (1) well-fed and (2) malnourished.

As recommended by the GLIM, the term “malnutrition” was used in lean people but without a history of cancer, and in cancer patients the term “cancer cachexia” [3]. Biochemical markers of nutritional status (total protein, albumin, and absolute lymphocyte number) of patients with CRC were compared with reference values determined by our laboratory. According to the SGA scale, we rated the most malnourished people, which is why we used it as a reference to assess the effectiveness of other methods.

### 2.4. Statistical Analyses

Data was analyzed using Statistica version 13.3 (StatSoft Inc., 2019). The value of *p* < 0.05 was considered relevant for all analyses. The results were expressed as mean ± SD.

The normality of variables was assessed using visual inspection of histograms and the measures of skewness and kurtosis as well as Shapiro–Wilk tests. The statistical significance of differences between groups was verified using Student's *t*-test and Mann–Whitney *U* test, and the sensitivity and specificity of the parameters tested were determined using area under the ROC curve. In the case of normal distribution of variables, the Student *t*-test was used, and in the absence of normal distribution, Mann–Whitney's *U* nonparametric test was used.

ROC curves were constructed that displayed the sensitivity vs. 1 −  specificity values. Predictive capacity was quantified by the AUC ROC curve. Curve analysis (ROC) was used to determine the FMI, FFMI, SMMI, FM/FFM, and SMMI cutoff points separating CRC patients from the control group and to identify malnutrition. Youden's index (sensitivity % + specificity % − 100%) was used to assess the correctness of the diagnostic test identifying malnutrition and to determine optimal cutoff points.

## 3. Results

### 3.1. Comparison of BIA Parameters of Men vs. Women and Persons below vs. above 65 y in the Control Group

There were significant differences in body composition between women and men in SMMI and FFMI; only PA did not differ significantly. Extracellular Water/Total Body Water (ECW/TBW) and reactance (Xc) in the elderly were higher (*p* < 0.05) and phase angle (PA) lower (<0.05) than in the younger subjects. The results of this comparison justified the need for a separate analysis of BIA parameters of women and men, but without considering the age difference of the examined patients.

### 3.2. AUC ROC Curve to Determine Cut-Off Points for BIA Parameters Relative to SGA, Which Distinguish between Well-Fed and Malnourished Study Participants

AUC ROC and Youden's index (I-Youden) were used to determine the cutoff points for BIA parameters distinguishing well-nourished from undernourished subjects, and SGA was taken as a reference. AUC ROC for FFMI and SMMI (*p* < 0.01) in men and SMMI and ECW/TBW (*p* < 0.01) in women were statistically significant (Table 1).

### 3.3. Nutritional Status of Study Participants according to BMI, NRS 2002, BIA, and Biochemical Parameters Compared to SGA

Most patients with CC in the group with CRC were diagnosed according to SGA; therefore, SGA was taken as a reference point for the evaluation of effectiveness of the remaining methods. Among other methods, NRS 2002 had the highest sensitivity (96%) and specificity (58%) (Table 2).

**Figure 1:**
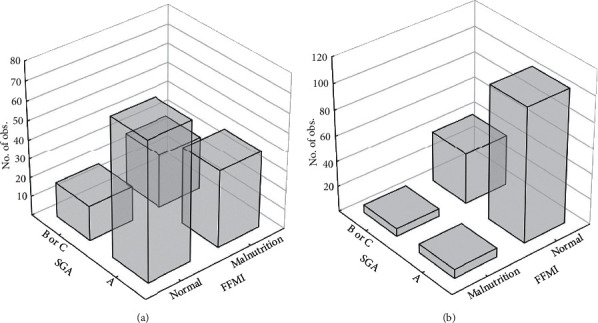
Comparison of nutritional status of CRC patients by cutoff points for FFMI according to Cederholm et al. and determined by us for the study participants. (a). FFMI according to Youden's test (female <16.2 kg/m^2^, male <19.4 kg/m^2^). Bivariate histogram SGA × FFMI (F <16.2, M <19.4). (b). FFMI according to Cederholm et al. (female <15 kg/m^2^, male <17 kg/m^2^). Bivariate histogram SGA × FFMI (F <15, M <17).

The positive prediction value (PPV) for FFMI (F-13%, M-14%) for the diagnosis of malnutrition according to Cederholm et al. (F <15 kg/m^2^, M <17 kg/m^2^) was lower (F-58%, M-64%) than for the cutoff points established in our study (F <16.2 kg/m^2^, M <19.4 kg/m^2^) (Table 2) (Figure 1).

In the control group, no undernourished persons were diagnosed according to SGA, NRS 2002, and biochemical parameters, but 8 (9%) undernourished persons were diagnosed according to FFMI Cederholm et al. and 34 (39%) persons according to FFMI cutoff points established by us.

### 3.4. Nutrition Status of All Study Participants according to BMI Criteria

In the control group, 60% were overweight or obese and in the CRC group 51%. Only 2 persons (3%) with CRC were diagnosed with CC according to BMI criterion (Table 3). Low PPV (4%) indicates extremely poor ability of BMI to diagnose CC (Table 2).

### 3.5. BIA Patients with CRC vs. Control Group by Gender

Control men had FFMI, SMMI higher (*p* < 0.001) and ECW/TBW, R, and Xc lower (*p* < 0.05) than men with CRC. Control women had Xc and PA (*p* < 0.05) lower than women with CRC (Table 4).

PA is a widely recognized indicator of malnutrition. Correlation coefficients between FFMI, SMMI, ECW/TBW, and PA of women and men with control were statistically significant (*p* < 0.05), but in men with CRC were not significant (Table 5).

The values of correlation coefficients of SMMI (*r* = 0.397 vs. *r* = 0.636) and ECW/TBW (*r* = −0.664 vs. *r* = −0.917) to PA of women with CRC differed significantly from those of women with control, but there was no significant difference between the correlations of FMI and PA (*r* = 0.147 vs. *r* = 0.031).

**Figure 2:**
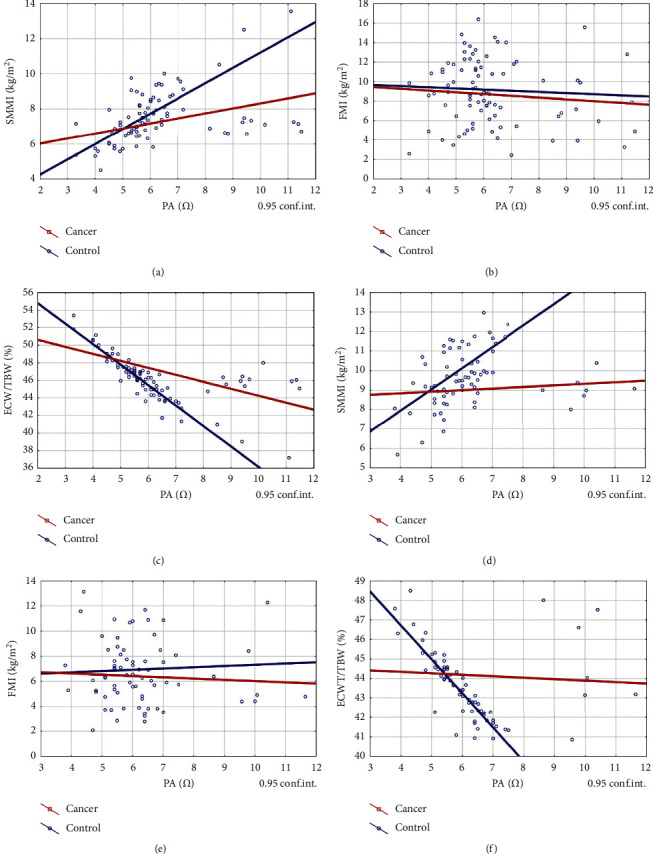
Correlations of SMMI, FMI, and ECW/TBW of control and CRC patients. Female: (a) SMMI, (b) FMI, and (c) ECW/TBW. Male: (d) SMMI, (e) FMI, and (f) ECW/TBW. (a) Female cancer vs. control. PA vs. SMMI. Correlation—control: *r* = 0.63591, cancer: *r* = 0.39679. (b) Female cancer vs. control. PA vs. FMI. Control: *r* = −0.0307, cancer: *r* = −0.1473. (c) Female cancer vs. control. PA vs. ECW/TBW. Control: *r* = −0.9173, cancer: *r* = −0.6635. (d) Male cancer vs. control. PA vs. SMMI. Correlation—control: *r* = 0.6117, cancer: *r* = 0.1553. (e) Male cancer vs. control. PA vs. FMI. Correlation—control: *r* = 0.03910, cancer: *r* = −0.0694. (f) Male cancer vs. control. PA vs. ECW/TBW. Correlation—control: *r* = −0.919, cancer: *r* = −0.079.

The values of SMMI (*r* = 0.155 vs 0.612) and ECW/TBW (*r* = −0.079 vs. *r* = −0.919) correlation coefficients to PA of men with CRC differed significantly from those of men from control, but there was no significant difference between FMI (*r* = −0.069 vs. *r* = 0.039) and PA (Figure 2). This can lead to a conclusion that PA varied with SMMI and ECW/TBW and not with FMI.

### 3.6. BIA of Patients with CRC Stage I vs. II–IV and Stage I-II vs. III-IV

Men in stage I of CRC had larger (*p* < 0.05) BMI, FMI, SMMI, ECW/TBW than men in stages II–IV, but the FM/FFM and SMM/FM ratios did not differ. The FM/FFM ratio of men in stage I-II of CRC was higher (*p* < 0.05) and SMM/FM was lower (*p* < 0.05) than in men in stage III-IV, and the remaining BIA parameters did not differ significantly.

Women in stage I of CRC had higher (*p* < 0.05) BMI, FMI, FFMI, FM/FFM, and SMM/FM and R lower (*p* < 0.05) than women in stages II–IV. When comparing women in stages I-II with women in stages III-IV, only FM (*p* < 0.05) and R (*p* < 0.05) were higher (Table 6).

### 3.7. AUC ROC and Cutoff Points for BIA Parameters of Stage I vs. II–IV and Stages I-II vs. III-IV CRC Patients

Male AUC ROC for FMI (0.857; *p* < 0.001) and SMMI (0.755; *p* < 0.05) in stage I vs. stages II–IV CRC were significantly higher than in stages I-II vs. III-IV (FMI—0.594; *p*—ns and SMMI—0.657; *p*—ns); thus, the cutoff points according to Youden's *J* statistic (FMI—6.4 kg/m^2^, SMMI—9.4 kg/m^2^) should distinguish them more effectively.

Women's AUC ROC for FMI (0.800; *p* < 0.01) and SMMI (0.723; *p* < 0.01) with CRC in stage I vs. II–IV were much larger than in stages I-II vs. III-IV (FMI—0.739; *p* < 0.01 and SMMI—0.561; *p*—ns); thus, the cutoff points according to Youden's *J* statistic (FMI—11.3 kg/m^2^, SMMI—7.3 kg/m^2^) should distinguish them more effectively.

**Figure 3:**
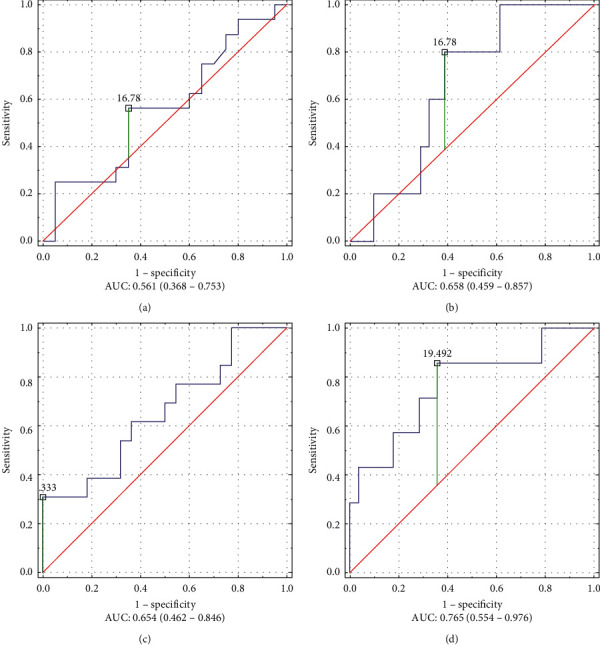
Comparison of AUC ROC for FFMI patients with CRC stages I-II vs. III-IV and stage I vs. II–IV. (a) Female in stages I-II vs. III-IV. FMMI. Female CRC, stages I-II vs. III-IV. Youden's index = 0.21, proposed cutoff point: 16.78. (b) Female in stage I vs. III-IV FMMI. Female CRC, stage I vs. II–IV. Youden's index = 0.41, proposed cutoff point: 16.78. (c) Male in stages I-II vs. III-IV. FFMI. Male CRC, stages I-II vs. III-IV. Youden's index = 0.31, proposed cutoff point: 21.33. (d) Male in stage I vs. III-IV. FFMI. Male CRC, stage I vs. II–IV. Youden index = 0.50, proposed cutoff point: 19.49.

AUC ROC for FFMI (male—0.765, female—0.658) and SMMI (male—0.755, female—0.723) in stage I vs. II–IV patients were larger than AUC ROC for FFMI (male—0.654, female—0.561) and SMMI (male—0.657, female—0.561) in stage II vs. III-IV patients. Therefore, the cutoff points for FFMI (male—19.5 kg/m^2^ and female—16.8 kg/m^2^) and SMMI (male—9.4 kg/m^2^ and female—7.3 kg/m^2^) distinguish more effectively patients in stage I vs. II–IV than the cutoff points established to distinguish stage I-II vs. III-IV (Figure 3).

## 4. Discussion

The analysis of BIA results confirmed significant differences between the body composition of the examined women and men. This confirms the need to consider gender when analyzing BIA results. Due to age-related sarcopenia, a man loses about 1-2% of SMM/year from the age of 50 years and up to 3% every year after 60 years of age [16, 17]. However, in the control group, age-related differences included only Xc and PA and fluid distribution (ECW/TBW). We believe that the lack of differences in other parameters resulted from the exclusion of people below 50 years of age from the study.

To differentiate well-nourished from undernourished participants, the AUC ROC analysis was performed, and cutoff points were determined according to the Youden index (Table 1). The SGA scale was the most effective in recognizing malnutrition, so we took it as a reference point for other methods of nutritional status assessment (Table 2). According to SGA, NRS 2002, BMI, and biochemical parameters, undernourished individuals were not recognized in the control group, but malnutrition was diagnosed in 8 (9%) people according to FFMI proposed by Cederholm et al. and in 34 (39%) people for FFMI established by us (Table 3) (Figure 1). Such many false positive results in the control group undermines the reliability of the assessment of nutritional status according to FFMI.

Due to the prevalence of overweight and obesity, many CRC patients are still overweight despite WL. The analysis of the results of the study participants confirmed the validity of this statement. According to the BMI criteria, 60% of the control group were overweight or obese and 51% in the CRC group. CC was found only in 2 (3%) patients with CRC (Table 3). A low predictive positive value (4%) indicates low ability of BMI to recognize CC in the early stages (Table 2). The effectiveness of biochemical parameters for the diagnosis of malnutrition in patients with CRC was also exceptionally low.

The definition of CC emphasizes that the loss of SMM may or may not be accompanied by the loss of FM. To determine the changes of SMM and FM in CRC, we compared the BIA parameters of patients with CRC and controls. Men with CRC had lower FFMI, SMMI (*p* < 0.001) and ECW/TBW, R, and Xc higher (*p* < 0.05) than men with controls (Table 4). There was no significant difference between parameters of women with CRC and controls; only Xc and PA were higher in women with CRC (*p* < 0.05). This is intriguing, compared to the results of many other studies which have shown that PA was lower in both women and men with cancer, respectively, in the stage of CC.

The correlation coefficients of FFMI (*p* < 0.05), SMMI (*p* < 0.05), and ECW/TBW (*p* < 0.001) to PA were statistically significant in women and men with control and in women with CRC but were not significant in men with CRC (Table 5). This suggests that WL in women is different than in men with CC. The correlation coefficients of SMMI and ECW/TBW to PA of patients with CRC differed from control, but the correlation coefficients of FMI to PA were remarkably similar (Figure 2). From this, we can conclude that the value of PA varies with SMMI and ECW and not with FFMI. This is consistent with the claim that in CC the leading element is the loss of SMM with or without loss of FM.

CC is synchronously deepening to the progression of cancer, so it is interesting to compare the BIA parameters of patients in stage I vs. II–IV CRC and stages I-II vs. III-IV (Table 6). Here, it is worth noting the indices weight/gain^2^, which are more eloquent than the component weight alone, as is the case with BMI.

When comparing men with CRC in stages I-II vs. III-IV, FFMI and SMMI do not differ significantly; only FMI is higher (*p* < 0.05). Men in stages II–IV have smaller FFMI, SMMI, and ECW/TBW (*p* < 0.05) than in stage I. This shows that men start losing FM (*p* < 0.001) in stage I of CRC and SMM (*p* < 0.05) later in stage II.

When comparing BIA parameters of women in stages I-II vs. III-IV of CRC, FFMI and FMI values do not differ significantly, but when comparing stage I vs. II–IV, FM, FMI, and FFMI values in stages II–IV are significantly lower (*p* < 0.05) than in stage I. The effect of FM decreases in II, and in subsequent stages of CRC, there is a decrease in FM/FFM ratio and an increase in SMM/FM (*p* < 0.05).

AUC ROC is higher when comparing the BIA parameters of patients in stages I-II vs. III-IV of CRC than when comparing the BIA parameters of patients in stage I vs. II–IV. To illustrate the difference between FFMI in different stages of CC, AUC ROC is presented in graphical form (Figure 3).

## 5. Conclusions

Nutrition assessment should be carried out before the start of treatment, as many patients suffer from weigh loss before the diagnosis of cancer. SGA and NRS 2002 are effective in detecting malnutrition, but do not determine the body composition of oncological patients, which changes according to the stage of CC. The ability of BIA to detect malnutrition is less than SGA and NRS 2002 BIA, but it effectively differentiates the body composition of patients according to the stage of CRC and CC. Many factors influence the body composition, thus establishing universal cutoff points for BIA parameters that effectively differentiate CC stage in patients with CRC is not possible. Therefore, BIA, as a method not superior to easy-to-use SGA and/or NRS 2002, should not be performed to diagnose cancer cachexia in colorectal cancer patients. However, it is possible to periodically repeat and compare BIA results in the same patient after the diagnosis of cancer. This approach can provide objective data on the dynamics of CC and on the effectiveness of oncological therapy and nutritional intervention.

## Figures and Tables

**Table 1:** AUC ROC and cutoff points of BIA parameters in relation to SGA to distinguish undernourished women and men from well-nourished women and men.

Sex	Variables	AUC (95% Cl)	*p*	I-Youden	Cutoff point
*Male*	FMI (kg/m^2^)	0.68 (0.538–0.822)	<0.01	0.38	5.65
	FFMI (kg/m^2^)	0.732 (0.615–0.849)	<0.01	0.4	19.4
	SMMI (kg/m^2^)	0.751 (0.64–0.861)	<0.01	0.45	9.9
	ECW/TBW (%)	0.411 (0.268–0.554)	ns	0.05	46
	PA (Ω)	0.624 (0.471–0.777)	ns	0.29	5.4

*Female*	FMI (kg/m^2^)	0.525 (0.392–0.658)	ns	0.16	12
	FFMI (kg/m^2^)	0.589 (0.455–0.723)	ns	0.17	16.2
	SMMI (kg/m^2^)	0.682 (0.549–0.815)	<0.01	0.35	6.62
	ECW/TBW (%)	0.332 (0.373–0.721)	<0.01	0.03	41
	PA (Ω)	0.547 (0.373–0.721)	ns	0.39	5.2

**Table 2:** Sensitivity and specificity of NRS 2002, BMI, FFMI, and biochemical parameters in the detection of malnutrition in CRC patients in relation to SGA.

	Cutoff point	SE (%)	SP (%)	PPV (%)	NPV (%)
NRS 2002	3 points	96	58	63	96
BMI	18.5 (kg/m^2^)	100	35	4	100
FFMI					
Female	15 (kg/m^2^)	75	34	13	92
Male	17 (kg/m^2^)	100	39	14	100
Female	16.2 (kg/m^2^)	88	50	58	83
Male	19.4 (kg/m^2^)	78	50	64	67
Protein	62 (g/l)	64	36	21	79
Albumin	32 (g/l)	100	38	7	100
Lymphocytes	1500 (10^9^/l)	72	44	59	58
CRP	5 (mg/l)	55	26	35	44

SE—sensitivity, SP—specificity, PPV—positive predicted values, and NPV—negative predicted values.

**Table 3:** Nutrition status of study subjects according to BMI criteria in relation to SGA.

		Overweight, *n* (%)	Malnutrition, *n* (%)	Normal, *n* (%)	Total, *n*
*Cancer*	Together	36 (51)	2 (3)	32 (46)	70
	Female	19 (53)	1 (3)	16 (44)	36
	Male	17 (50)	1 (3)	16 (47)	34

*Control*	Together	53 (60)	0 (0)	35 (40)	88
	Female	24 (48)	0 (0)	26 (52)	50
	Male	29 (76)	0 (0)	9 (24)	38

*All*	Together	89 (56)	2 (1)	67 (42)	158
	Female	43 (50)	1 (1)	42 (49)	86
	Male	46 (64)	1 (1)	25 (35)	72

**Table 4:** Comparison of BIA parameters of patients with CRC vs. control including gender difference.

Variable	Male		Female			
	Cancer	Control	*p*	Cancer	Control	*p*
	Mean ± SD	Mean ± SD		Mean ± SD	Mean ± SD	
FMI (kg/m^2^)	6.4 ± 2.8	6.9 ± 2.3	ns#	8.6 ± 3.1	9.2 ± 3.5	ns#
FFMI (kg/m^2^)	19.3 ± 2.0	21.1 ± 2.4	<0.001#	16.7 ± 2	16.6 ± 1.8	ns#
SMMI (kg/m^2^)	9.0 ± 1.0	10.1 ± 1.5	<0.001^*∗*^	7.4 ± 1.8	7.6 ± 1.2	ns^*∗*^
FM/FFM (%)	32.7 ± 13.4	32.7 ± 10.1	ns#	52.2 ± 19.4	55.8 ± 20.1	ns#
SMM/FM	1.66 ± 0.64	1.61 ± 0.54	ns#	1.05 ± 0.75	1.00 ± 0.66	ns#
ECW/TBW (%)	44.2 ± 1.8	43.2 ± 1.6	<0.05^*∗*^	46.8 ± 3	45.9 ± 2.3	ns^*∗*^
VAT (L)	3.2 ± 2.0	4.1 ± 1.8	ns#	1.6 ± 0.9	1.7 ± 0.8	ns#
R (Ω)	463.4 ± 58.8	429.2 ± 72.5	<0.05	527.8 ± 62.1	533.2 ± 60.8	ns#
Xc (Ω)	52.9 ± 17.1	44.6 ± 7.3	<0.05	61.3 ± 23	54 ± 9	<0.05
PA (Ω)	6.5 ± 1.9	6.0 ± 0.9	ns#	6.8 ± 2.5	5.8 ± 0.9	<0.05

^*∗*^Student's *t*-test. ^#^Mann–Whitney *U* test. ns—nonsignificant.

**Table 5:** Correlations between BIA parameters in relation to PA for women with CRC and control and for men with CRC and control.

Variable	Female				Male			
	Cancer		Control		Cancer		Control	
	To PA, *r*	*p*	To PA, *r*	*p*	To PA, *r*	*p*	To PA, *r*	*p*
FMI (kg/m^2^)	−0.147	ns	−0.031	ns	−0.069	ns	0.039	ns
FFMI (kg/m^2^)	0.347	*p* < 0.05	0.350	*p* < 0.05	−0.045	ns	0.498	*p* < 0.001
SMMI (kg/m^2^)	0.397	*p* < 0.05	0.636	*p* < 0.001	0.155	ns	0.612	*p* < 0.001
FM/FFM (%)	−0.222	ns	−0.137	ns	−0.079	ns	−0.145	ns
SMM/FM	0.341	*p* < 0.05	0.236	ns	0.031	ns	0.314	ns
ECW/TBW (%)	−0.664	*p* < 001	−0.917	*p* < 0.001	−0.079	ns	−0.919	*p* < 0.001

**Table 6:** Men and women with CRC in stages I-II vs. stages III-IV and stage I vs. stages II–IV.

Sex	Variable	Stage I-II	Stage III-IV	*p*	Stage I	Stage II–IV	*p*
		Mean ± SD	Mean ± SD		Mean ± SD	Mean ± SD	
*Male*	FM (kg)	21.8 ± 9.4	17.8 ± 8.2	ns^*∗*^	28.2 ± 8	17.0 ± 7.5	<0.001^*∗*^
	FMI (kg/m^2^)	7.1 ± 3.1	5.9 ± 2.6	<0.05#	9.3 ± 2.6	5.6 ± 2.4	<0.05#
	FFMI (kg/m^2^)	20.1 ± 2.3	18.9 ± 1.6	ns#	21.0 ± 2.6	18.9 ± 1.6	<0.05#
	SMM (kg)	29.1 ± 4.8	26.2 ± 3.4	<0.05^*∗*^	29.7 ± 4.3	26.7 ± 4	ns^*∗*^
	SMMI (kg/m^2^)	9.4 ± 1	8.8 ± 1	ns^*∗*^	9.7 ± 1.1	8.9 ± 0.9	<0.05^*∗*^
	FM/FFM (%)	34.8 ± 13.7	31.4 ± 13.4	<0.05#	44.3 ± 11.9	29.7 ± 12.3	ns#
	SMM/FM	1.53 ± 0.55	1.75 ± 0.69	<0.05#	1.11 ± 0.27	1.81 ± 0.63	ns#
	ECW/TBW (%)	44.7 ± 2	43.8 ± 1.6	ns^*∗*^	46.1 ± 1.5	43.7 ± 1.5	<0.05^*∗*^
	R (Ω)	432.6 ± 59.5	482.4 ± 50.9	ns#	405.2 ± 55.1	478.4 ± 50.4	ns#
	Xc (Ω)	54.1 ± 19.4	52.2 ± 15.9	ns#	50.1 ± 17	53.7 ± 17.3	ns#
	PA (Ω)	7.1 ± 2.3	6.2 ± 1.7	ns#	7.1 ± 2.4	6.4 ± 1.8	ns#
*Female*	FM (kg)	25.5 ± 7.8	19.3 ± 6.2	<0.05^*∗*^	30.5 ± 6	20.7 ± 6.9	<0.05^*∗*^
	FMI (kg/m^2^)	10.0 ± 3.1	7.4 ± 2.6	ns#	11.5 ± 2.8	8.1 ± 2.9	<0.05#
	FFMI (kg/m^2^)	16.8 ± 1.7	16.6 ± 2.2	ns#	17.3 ± 1.1	16.6 ± 2.1	<0.05#
	SMM (kg)	19.2 ± 4.6	19.4 ± 6	ns^*∗*^	21.1 ± 3.4	19 ± 5.6	ns^*∗*^
	SMMI (kg/m^2^)	7.5 ± 1.8	7.3 ± 1.9	ns^*∗*^	7.8 ± 0.6	7.3 ± 1.9	ns^*∗*^
	FM/FFM (%)	60.6 ± 19.2	45.5 ± 17.1	ns#	66.7 ± 17	49.8 ± 18.9	<0.05#
	SMM/FM	0.91 ± 0.7	1.17 ± 0.78	ns#	0.72 ± 0.21	1.11 ± 0.79	<0.05#
	ECW/TBW (%)	46.9 ± 3.1	46.6 ± 3	ns^*∗*^	46.8 ± 1.9	46.8 ± 3.2	ns^*∗*^
	R (Ω)	514.2 ± 56.8	538.6 ± 65.4	<0.05#	488.9 ± 44.9	534.0 ± 62.7	<0.05#
	Xc (Ω)	55.7 ± 22.8	65.8 ± 22.7	ns#	52.7 ± 16.2	62.67 ± 23.8	ns#
	PA (Ω)	6.5 ± 2.4	7.1 ± 2.7	ns#	6.2 ± 2	6.9 ± 2.6	ns#

^*∗*^Test *t*. ^#^*U* Mann–Whitney.

## Data Availability

The original data used to support the findings of this study are available from the corresponding author upon request.
